# Acoustic structure of male loud-calls support molecular phylogeny of Sumatran and Javanese leaf monkeys (genus *Presbytis*)

**DOI:** 10.1186/1471-2148-12-16

**Published:** 2012-02-06

**Authors:** Dirk Meyer, John K Hodges, Dones Rinaldi, Ambang Wijaya, Christian Roos, Kurt Hammerschmidt

**Affiliations:** 1 Reproductive Biology Unit, German Primate Center, Göttingen, Germany; 2 Göttingen Center for Biodiversity and Ecology, Göttingen, Germany; 3 Department of Forest Resources Conservation and Ecotourism, Bogor Agricultural University, Bogor, Indonesia; 4 WWF Kalimantan Tengah, Palangkaraya, Indonesia; 5 Gene Bank of Primates and Primate Genetics Laboratory, German Primate Center, Göttingen, Germany; 6 Cognitive Ethology Laboratory, German Primate Center, Göttingen, Germany

## Abstract

**Background:**

The degree to which loud-calls in nonhuman primates can be used as a reliable taxonomic tool is the subject of ongoing debate. A recent study on crested gibbons showed that these species can be well distinguished by their songs; even at the population level the authors found reliable differences. Although there are some further studies on geographic and phylogenetic differences in loud-calls of nonhuman primate species, it is unclear to what extent loud-calls of other species have a similar close relation between acoustic structure, phylogenetic relatedness and geographic distance. We therefore conducted a field survey in 19 locations on Sumatra, Java and the Mentawai islands to record male loud-calls of wild surilis (*Presbytis*), a genus of Asian leaf monkeys (Colobinae) with disputed taxanomy, and compared the structure of their loud-calls with a molecular genetic analysis.

**Results:**

The acoustic analysis of 100 surili male loud-calls from 68 wild animals confirms the differentiation of *P.potenziani, P.comata, P.thomasi *and *P.melalophos*. In a more detailed acoustic analysis of subspecies of *P.melalophos*, a further separation of the southern *P.m.mitrata *confirms the proposed paraphyly of this group. In concordance with their geographic distribution we found the highest correlation between call structure and genetic similarity, and lesser significant correlations between call structure and geographic distance, and genetic similarity and geographic distance.

**Conclusions:**

In this study we show, that as in crested gibbons, the acoustic structure of surili loud-calls is a reliable tool to distinguish between species and to verify phylogenetic relatedness and migration backgrounds of respective taxa. Since vocal production in other nonhuman primates show similar constraints, it is likely that an acoustic analysis of call structure can help to clarify taxonomic and phylogenetic relationships.

## Background

Langurs of the Asian colobine genus *Presbytis *(surilis) are exclusively arboreal animals, which inhabit tropical rainforest habitats of Sundaland, i.e., the Malay peninsula and the western Indo-Malay archipelago, comprising of Sumatra, Borneo, Java, the Mentawai islands and some smaller interjacent islands [1]. Mainly driven by Sundaland's dramatic geological and climatic changes during the past million years, the genus has undergone an extensive radiation [2]. With more than 50 described color variants [3,4], *Presbytis *is one of the most diverse primate genera among Old World monkeys.

Like many other primate species, surilis emit loud, conspicuous vocalizations termed loud-calls or long-distance calls. In contrast to *Presbytis*, gibbon loud-calls have a well-adapted acoustic structure [5,6]; with an energy concentration in single frequency bands, a slow modulation of song elements and a transmission range adjusted to the frequency window of rainforest conditions, their songs can be heard over several miles [7,8]. Although less well optimized, loud-calls produced by other nonhuman primate species, such as howler monkeys [9] or surilis [10], also exhibit adaptations for long-distance transmission. Loud-calls can have a variety of different functions; they may be used to defend resources, to compete for mates, to mediate intergroup spacing and to promote intragroup cohesion [9,11,12]. In those species in which the structure of loud-calls is well adapted to long-distance transmission, they function predominantly to mark and defend territories.

Although there is general agreement that loud-calls may also serve as phylogenetic traits, systematic studies comparing call structure and genetic relatedness are rare. Amongst gibbons, structural differences are routinely used as a taxonomic tool [13,14]. In a recent study on crested gibbons carried out in 24 different locations in Vietnam, Laos and Cambodia, Thinh and colleagues [15] combined a molecular genetic analysis with an acoustic analysis and showed that song structure alone can be used to distinguish the different species. Based on call structure, the authors were also able to distinguish single populations and support not only their phylogentic relatedness, but also their proposed geographic origins. Comparable studies in other nonhuman primates are lacking. However, single studies on loud-calls of orangutans [16], Thomas langurs [17], chimpanzees [18], black-and-white colobus monkeys [19] or sportive lemurs [20] revealed geographic or genetic related differences in the structure of loud-calls of these species. Some previous studies proposed that loud-calls of surilis could be a useful tool to characterize phylogenetic relatedness [21-23]. According to these studies, the Sumatran surilis were divided into the species *P.melalophos, P.femoralis, P.thomasi *[21-23] and *P.potenziani *[21,23], and Wilson and Wilson [23] proposed a successive invasion of Sumatra, Borneo and the Mentawai islands from the Asian mainland. However, all these studies are only based on phonetic descriptions of loud-calls and did not make a systematic analysis of the acoustic structure or a direct comparison between acoustic structure and genetic relatedness.

Here we combine the results of the most comprehensive molecular genetic study on leaf monkeys of the genus *Presbytis *currently available [24] with a systematic field survey in which the loud-calls of *P.potenziani siberu, P.comata comata, P.thomasi *and the four subspecies of *P.melalophos *(*melalophos, mitrata, bicolor *and *sumatrana*) were recorded [3]. Previous classifications and phylogenies of *Presbytis *were mainly based on behavioral and anatomical features, in particular coat coloration [1,3,4,22,25-31], while genetic studies are extremely limited [24,32-34]. In our recent study [24], mitochondrial DNA was used to propose a revision of Groves' classification [3] suggesting species status for the four subspecies of *P.melalophos *and also for both subspecies of *P.comata *and *P.potenziani*. However, for convenience we use here the classification of Groves [3].

Since surilis intensively responds to stranger call playbacks [35], we used a playback design in order to collect vocalization data under comparable conditions. We hypothesized that, similar to crested gibbons, structural differences in *Presbytis *loud-calls reflect phylogenetic relationships and can support a revision of the current classification.

## Results

**Figure 1:**
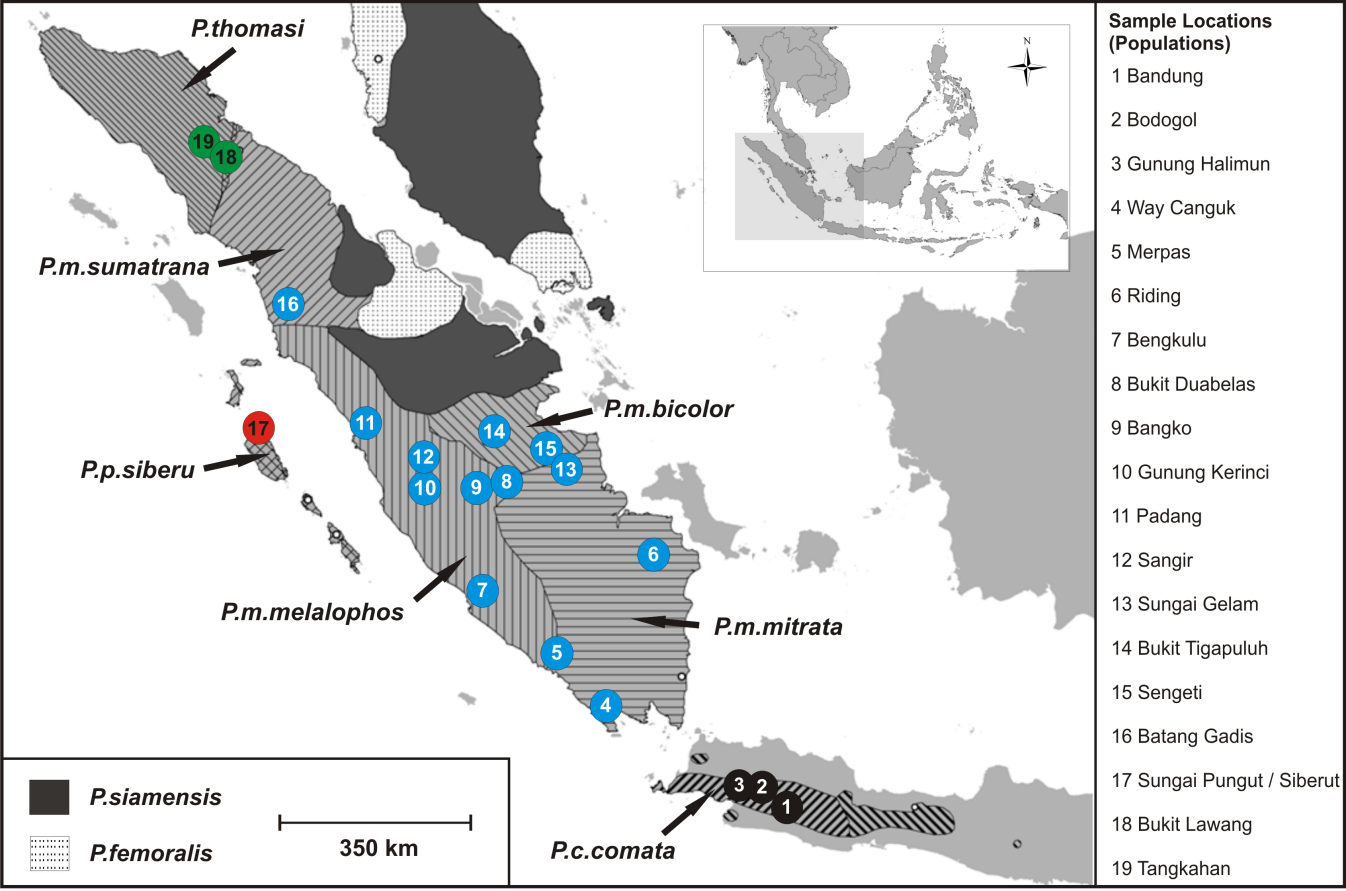
**Geographical distribution of *Presbytis *taxa on Sumatra, Java and the Malay peninsula**. Sampled taxa are labeled in the map. Hatched areas in the map indicate distribution ranges of respective taxa, colors indicate species and numbers indicate the origin of acoustic samples (populations).

In 2007 and 2008, we conducted field surveys in 19 locations (which resemble 19 populations) on Sumatra, Java and the Mentawai islands, and recorded male loud-calls of seven wild non-habituated *Presbytis *taxa (Figure 1). Included are *P.thomasi, P.potenziani siberu, P.comata comata *and all four subspecies of *P.melalophos *(*P.m.melalophos, P.m.mitrata, P.m.bicolor *and *P.m.sumatrana*). In total, we recorded more than 300 loud-calls of 68 male individuals.

In response to the playbacks, males often responded several times, but only one call of this bout was used for the analysis (in total 100 calls). Counter calling males in general decreased the distance to the speaker, while females hid or disappeared. A further common response to playback treatments was alarm calling of group members and in addition juveniles often started to squeal [36]. Loud-calls were mostly accompanied by a jumping display.

### General differences in male loud-calls

**Figure 2:**
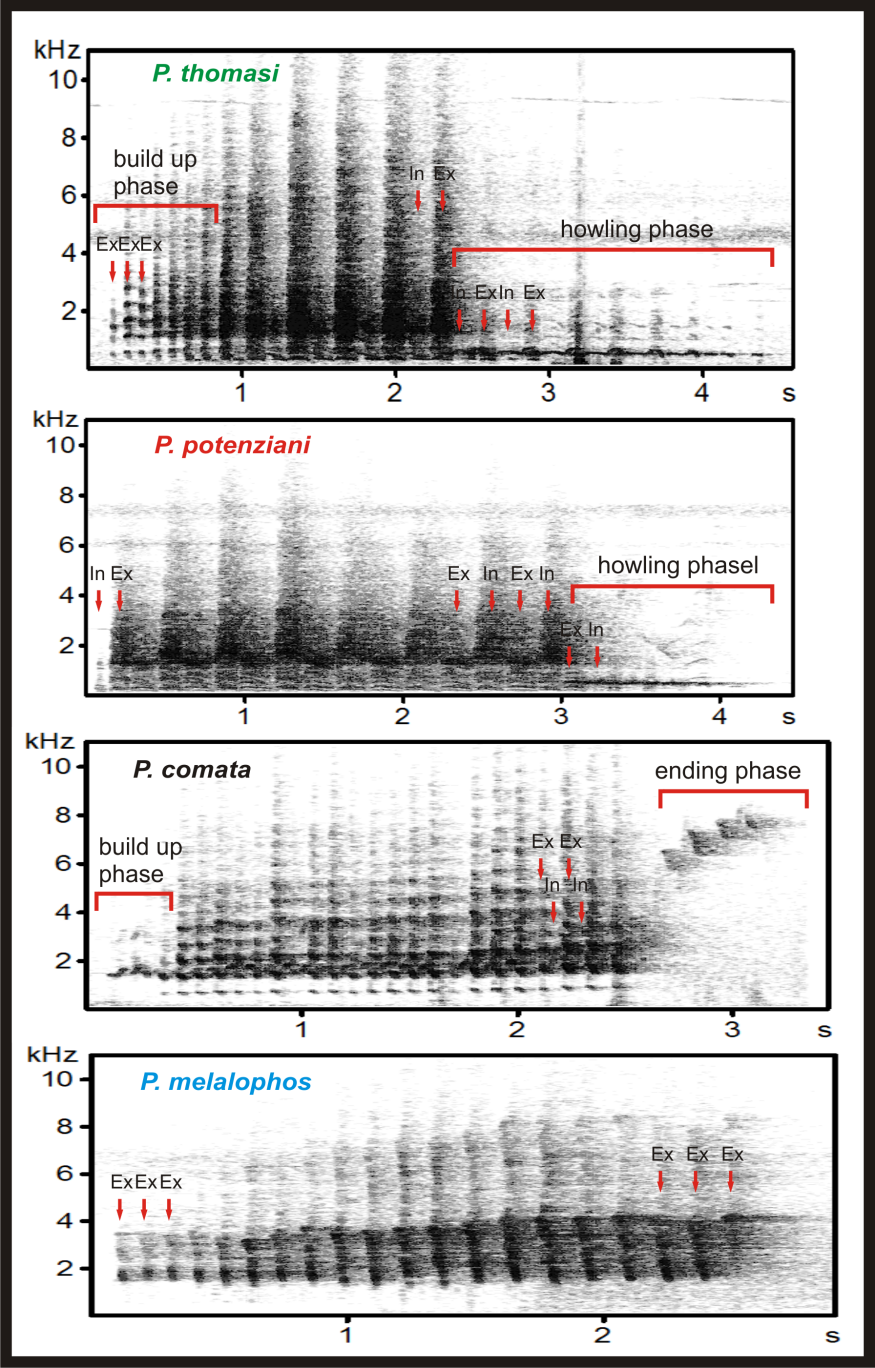
**Spectrograms of typical loud-calls of *P.thomasi, P.potenziani, P.comata *and *P.melalophos***.

*P.thomasi, P.potenziani, P.comata *and *P.melalophos *can be clearly identified by general acoustic characteristics in their call structure (Figure 2). In addition, species' calls are readily distinguished by ear, but *P.melalophos *subspecific differences are undetectable.

*P.thomasi *(n = 10) and *P.potenziani *(n = 9) calls start with coughing elements at the beginning and end with howling tonal phrases. These two parts include inhalation and exhalation elements. In *P.thomasi*, the initial coughing elements rise in crescendo and increase in volume (build-up phase). In *P. potenziani *(n = 9), the build-up phase is missing and the coughing elements are equally loud and noisy (Figure 2). Both loud-calls also differ in their mean duration with 3.58 s (SD = 0.35) for *P.thomasi *and 4.17 s (SD = 0.42) for *P.potenziani*. On the average, *P.potenziani *produces 28 elements (SD = 2) per call with a mean element frequency of 6.42 per second (SD = 0.56), while *P.thomasi *produces 30 elements (SD = 4) with a mean element frequency of 8.5 elements/s (SD = 1) (Figure 2). Detailed differences in the acoustic structure can be found in Additional File 1.

The typical *P.comata *call (n = 10) is characterized by a unique staccato-like sequence of 52 (SD = 8) alternating exhalation and inhalation elements (mean 18.20, SD = 1.91 elements/s). *P.comata *calls, with a mean call duration of 2.86 s (SD = 0.26), include a short build-up phase and an end-up phase, both with increasing loudness and frequency (Figure 2, Additional File 1).

**Figure 3:**
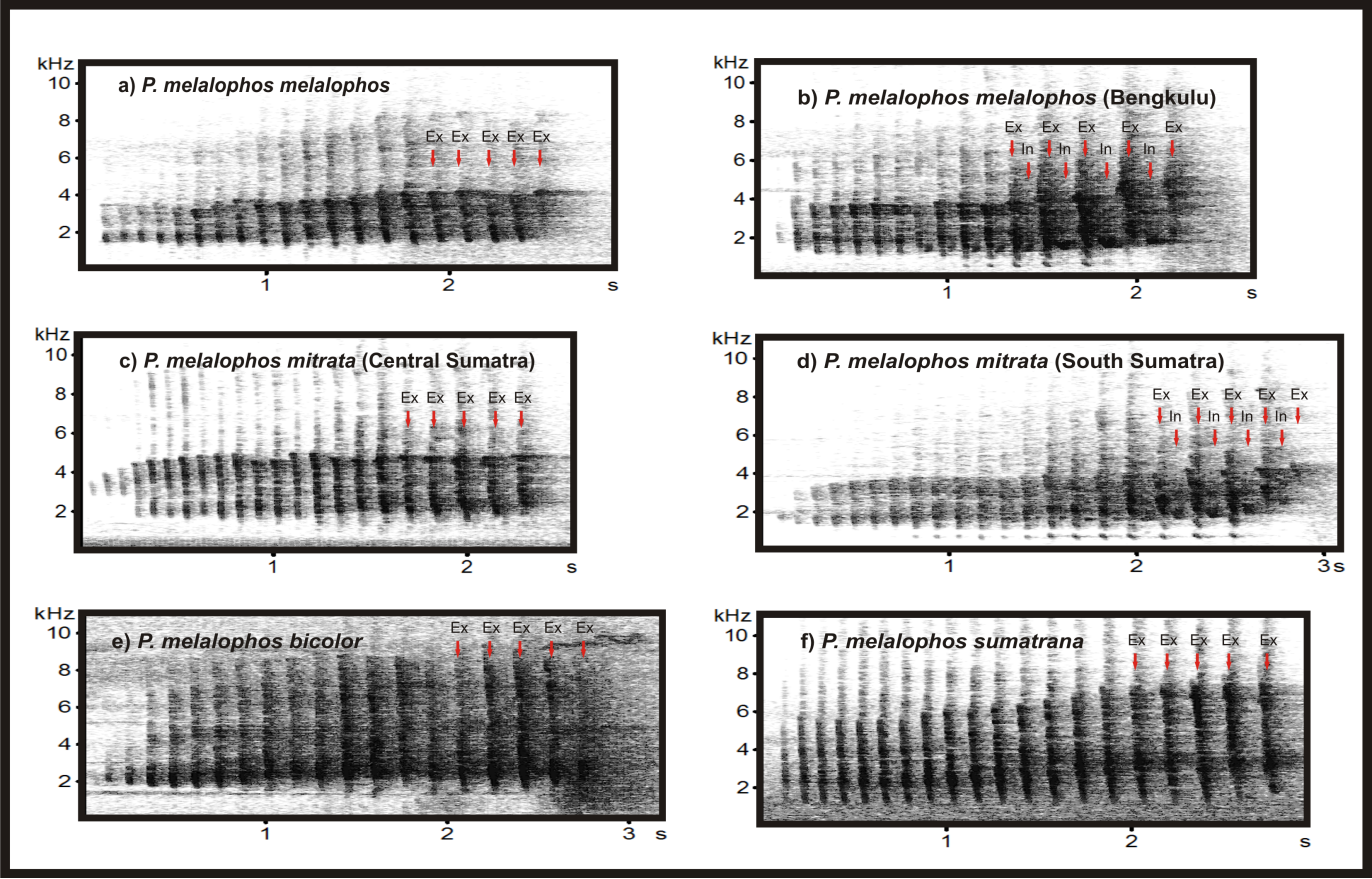
**Spectrograms of typical loud-calls of *P.melalophos *subspecies**.

Loud-calls of *P.m.bicolor *(n = 15), *P.m.sumatrana *(n = 9), the central Sumatran *P.m.mitrata *(n = 7) and *P.m.melalophos *(n = 26) from outside of Bengkulu, consist of a sequence of exhalation elements. An exception are the calls of *P.m.melalophos *from Bengkulu (n = 3) and the southern Sumatran *P.m.mitrata *(n = 12), which differ by producing alternating exhalation and inhalation elements at the end of the call (Figure 3). The mean duration of *P.melalophos *calls lies between 2.39 s (SD = 0.33) for *P.m.mitrata *and 2.53 s (SD = 0.40) for *P.m.sumatrana*. The mean frequency of produced elements lies between 10.85 elements/s, (SD = 2.36) for *P.m.mitrata *and 7.35 elements/s (SD = 0.4) for *P.m.bicolor *(Figure 3, Additional File 1).

### Subtle differences in male loud-calls

#### Result of the discriminant function analysis of all 19 populations (DFA1)

The DFA assigns 72% of the loud-calls (62% of cross-validated, chance level = 5.3%) to the original populations. In relation to taxon identity 83% of the cross-validated cases are correctly classified. Most misclassified cases are found between *P.melalophos *subspecies (Table 1).

**Table 1:** Classification results of the first and second DFA in relation to the taxon membership*

	*P.comata* n = 10 P 1-3	*P.m.mitrata* n = 19 P 4-6, 13	*P.m.melalophos* n = 29 P 7-12	*P.m.bicolor* n = 15 P 14-15	*P.m.sumatrana* n = 8 P 16	*P.potenziani* n = 9 P 17	*P.thomasi* n = 10 P 18-19
***P.comata*** I = 8	**100**						
***P.m.mitrata*** I = 13		**68/89**	26/11	6/0			
***P.m.melalophos ***I = 18		7/7	**79/76**	14/10	0/7		
***P.m.bicolor*** I = 13		7/0	20/7	**73/93**			
***P.m.sumatrana ***I = 5			12/25	0/12	**88/63**		
***P.potenziani*** I = 4						**100**	
***P.thomasi*** I = 7							**100**

*Relative predicted group membership in % (DFA 1/DFA 2), n = calls, P = population numbers (see also Figure 1), I = individuals.

**Figure 4:**
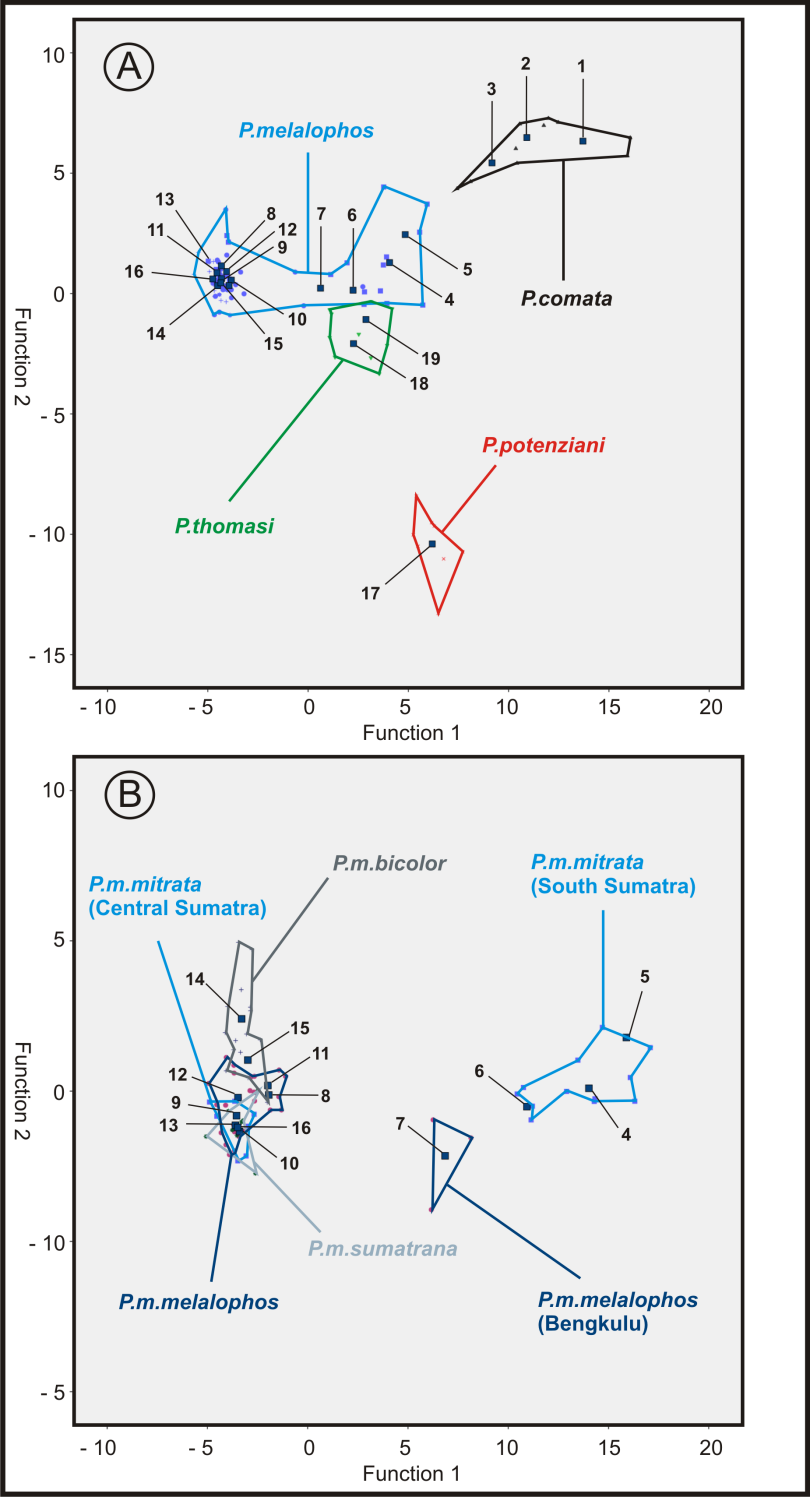
**Scattergram presenting the results of the DFA. A: Assignment of the four species *P.melalophos, P.comata, P.thomasi *and *P.potenziani*. B: Assignment of *P.melalophos *subspecies**. Rectangles indicate population centroids. Species are marked by color.

No misclassification can be found between the four *Presbytis *species, *P.comata *(populations 1-3), *P.melalophos *(populations 4-16), *P.potenziani *(population 17) and *P.thomasi *(populations 18-19). They form four well separated clusters with a correct assignment of 100% (Table 1, Figure 4A). Among the large *P.melalophos *cluster, one further sub-cluster is indicated, which includes *P.m.melalophos *from Bengkulu (population 7) and the southern Sumatran *P.m.mitrata *(populations 4-6). The scattergram (Figure 4A) shows the separation of the 19 populations according to the first and second discriminant function, explaining 56.3% and 26.7% of the total acoustic variation, respectively. The first discriminant function, which mainly represents the amount of inhalation elements, separates populations 1-3 from all others, while the second discriminant function, which represents rhythmical features, separates population 17 from all others. To focus on the *P.melalophos *cluster (populations 4-16), we conducted a second DFA.

#### Result of the discriminant function analysis of *P.melalophos *populations (DFA2)

The second DFA2 assigns 66.2% of the loud-calls (49.3% of cross-validated, chance level = 7.7%) to the original populations and establishes three distinct clusters (Figure 4B), separating the southern Sumatran *P.m.mitrata *(populations 4-6) and the *P.m.melalophos *from Bengkulu (population 7) from the remaining *P.melalophos *populations.

In relation to the taxon identity 89% *P.m.mitrata*, 76% *P.m.melalophos*, 93% *P.m.bicolor *and 63% *P.m.sumatrana *of the cross-validated population cases are correctly classified (Table 1). The scattergram (Figure 4B) shows the separation of the 13 populations according to the first and second discriminant function, explaining 92.9% and 3.8% of the total variation, respectively. The first discriminant function, which explains nearly all structural differences, represents the amount of inhalation elements, separates populations 4-6 from population 7, and the remaining populations. The second discriminant function mainly based on the minimum frequency of the call, indicates the separation of populations 14, 15 and 5 from population 7 and the lasting locations.

#### Phylogenetic relationships among *Presbytis *taxa based on acoustic data and comparison with molecular data

**Figure 5:**
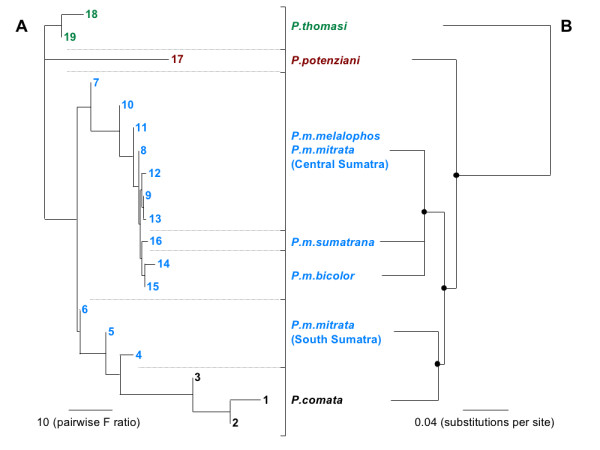
**(A) Neighbor-joining tree of *Presbytis *taxa based on the acoustic similarity matrix (F values) and (B) their phylogenetic relationships according to mitochondrial sequence data (redrawn from **[24]). In A, colored letters indicate species and numbers corresponding to sampling locations (see Figure 1). In B, branch lengths refer to those obtained from the Bayesian reconstruction in [24] and black dots on nodes indicate Bayesian posterior probabilities of > 0.96.

The vocal- (Figure 5A) and molecular-based phylogenies (Figure 5B) [24] are highly congruent. In both phylogenies, *P.thomasi, P.potenziani, P.melalophos *(excluding *P.m.mitrata *from South Sumatra) and *P.comata *+ *P.m.mitrata *from South Sumatra form four distinct clusters/lineages and indicate a similar branching pattern. Contrary to the molecular phylogeny, in the acoustic tree *P.m.mitrata *from South Sumatra (populations 4-6) does not form a monophyletic cluster, and *P.m.sumatrana *(population 16) and *P.m.bicolor *(populations 14-15) are nested within the cluster consisting of *P.m.melaophos *and *P.m.mitrata *from Central Sumatra (populations 7-13). This might be due to the subtle differences found in the vocal structure of respective populations.

#### Correlation between vocal structure, genetic and geographical distance

A Mantel test was performed to test the concordance between genetic distance, geographical distance and acoustic similarity. All populations where corresponding genetic data was available (N = 17) were included in the analysis (populations 3 [*P.comata*], populations 4,6,13 [*P.m.mitrata*], population 9 [*P.m.melalophos*], populations 14, 15 [*P.m.bicolor*], populations 17 [*P.potenziani*], populations 18, 19 [*P.thomasi*]; Figure 1). We found the highest significant correlations between vocal structure and genetic distance (P = 0.001, R_x, y _= 0.91), and lower significant correlations between genetic and geographical distance (P = 0.001, R_x, y _= 0.66), and geographical distance and vocal structure (P = 0.001, R_x, y _= 0.55).

## Discussion

Here we report significant differences between loud-call structures of *P.thomasi, P.potenziani, P.comata *and *P.melalophos*. Among the latter species a significant separation between the South Sumatran *P.m.mitrata *and the central Sumatran *P.m.mitrata*, as well as a further separation between *P.m.melalophos *from Bengkulu and the remaining *P.m.melalophos *populations, could be detected. The acoustic discrimination between *Presbytis *taxa was highly positively correlated with their genetic distance. In addition, we found substantial significant correlations between acoustic similarity and geographic distance and between genetic distance and geographic distance.

In our recent molecular genetic study [24] we suggested a paraphyly for *P.m.mitrata*, with the central Sumatran populations being closely related to *P.m.melalophos *and the South Sumatran populations forming a sister lineage to *P.comata *(Figure 5B). Our current findings on the acoustic structure of loud-calls strongly support these results.

*P.m.mitrata *was reported to inhabit the area southeast of the Batang Hari river, a large river in central Sumatra. In the west, this subspecies does not extend to the Bukit Barisan range, a mountain range on the western side of Sumatra [26], where *P.m.melalophos *occurs [3]. Our samples of the central Sumatran *P.m.mitrata *(population 13) derived from the above described northernmost distribution range of this subspecies, south of the Batang Hari river. Although much paler, the morphological appearance resembles the reddish *P.m.melalophos *more than the grayish white southern Sumatran *P.m.mitrata *(Additional File 2). Whether there might be a transition zone between *P.m.melalophos *and *P.m.mitrata *demands further research. It is highly likely that *P.m.melalophos *gradually intergrades with *P.m.mitrata*, as may be the case between *P.m.bicolor *and *P.m.melalophos *[26]. Our results, however, let us conclude that the central Sumatran *P.m.mitrata *population is the paler color variant of *P.m.melalophos*. Thus, the geographical distribution range of *P.m.melalophos *should be extended from the Bukit Barisan range eastwards towards Jambi. The southern Sumatran *P.m.mitrata *is genetically, morphologically and acoustically distinct from the remaining *P.melalophos *subspecies (see also Additional File 2). Therefore, if the Phylogenetic Species Concept [37,38] is applied, *P.m.mitrata *would be elevated to a monotypic species *P.mitrata *Eschscholtz, 1821 [39].

Among *P.m.melalophos *we found the calls from Bengkulu (population 7) forming a distinct cluster. Unfortunately, genetic data from Bengkulu are lacking, but acoustically, the call types were more closely related to the Southern *P.m.mitrata *mainly due to the presence of inhalation elements. Historically different color morphs of *P.m.melalophos *were described, all of which are currently classified as synonyms of *P.m.melalophos *[3]. These are a) the much less red and buffer variant from Bengkulu (*Simia melalophos *Raffles, 1821; syn. *flavimanus *Geoffroy, 1830), b) a foxy red northern form (*Presbytis nobilis *Gray, 1842) from Solok [4], c) a less reddish form from Padang (*Semnopithecus ferruginneus *Schlegel, 1876) and d) a golden buff variant (*Semnopithecus sumatranus var. aurata *Müller & Schlegel, 1841) from Gunung Talamau (ca. 150 km northwards from Padang) [3]. The great diversity of color morphs in *Presbytis*, in particular in *P.melalophos*, has caused much debate over the past decades. Coloration might indicate relatedness, but can often be misleading, in particular, when no broad geographic sampling is available. Our data point out that the taxonomic ranking of some of these historically described taxa possibly should be reconsidered. However, the loud-calls from population 7 are only derived from two individuals and genetic data are missing. Therefore, further molecular genetic and bio-acoustic research based on a broader sampling is needed to draw final conclusions. Of great interest are the acoustic data of the Bornean taxa, in particular data of *P.rubicunda*. Based on molecular genetic results *P.melalophos *is even polyphyletic since *P.rubicunda *is nested within the *P.m.sumatrana, P.m.bicolor, P.m.melalophos*/central Sumatran *P.m.mitrata *clade [24]. Previous studies already proposed a close affiliation of *P.rubicunda *and *P.melalophos *based on the red coat coloration [31] or in some aspects of behavior and vocalization [23]. If species status of *P.rubicunda *is retained, species status of *P.m.sumatrana, P.m.bicolor, P.m.melalophos *will be consequently warranted, otherwise *P.rubicunda *has to be assigned as a subspecies of *P.melalophos*.

The correlation between acoustic structure and genetic differences was higher than the correlation between acoustic structure and geographic distance. This pattern can be explained by the following proposed *Presbytis *migration pattern, which is largely in agreement with Wilson and Wilson [23]. The initial split in *Presbytis *occurred between *P.thomasi *and all other taxa, and *P.thomasi *colonized North Sumatra, which became isolated afterwards. The ancestor of the remaining taxa colonized first Borneo and later Sumatra. An early divergence of Bornean taxa is also supported by previous genetic studies [24,32-34]. Of the ancestral Sumatran stock, one lineage invaded the Mentawai islands (*P.potenziani*), the other split into the proto-*P.melalophos *lineage and into the southern *P.m.mitrata*/*P.comata *lineage (Figure 5). Although calls from *P.femoralis*/*P.siamensis *(eastern Sumatra, Asian mainland) are not analysed in our study, previous publications show similarities in call structures of *P.femoralis *and *P.thomasi *[40,41]. Our genetic study [24] shows that *P.femoralis *diverged relatively early from other lineages and, thus, the similar call structure of *P.femoralis *and *P.thomasi *might be a plesiomorphic feature. Up to this point the genetic, geographic and acoustic differences between populations increased. From this point onwards the geographic distances between populations decreased, because proto-*P.melalophos *subsequently transmuted into various present day subspecies, which were finally distributed across Sumatra as far as to the distribution range of *P.thomasi *in North Sumatra. Consequently, the geographic distance between *P.thomasi *and the remaining Sumatran populations decreased, while the genetic and the acoustic differences increased. Finally, the southern *P.m.mitrata/P.comata *lineage split into *P.m.mitrata *and *P.comata *that colonized Java. In this case we have a linear migration pattern and thus would expect a similar high correlation between acoustic structure, genetic and geographic distance, as it was currently shown in crested gibbons which are proposed to migrate in a linear fashion from North to South [15].

Surilis and gibbons are limited to rainforest habitats where the selection pressure forces an optimal adaptation of the structure of loud-calls for transmission over longer distances [5,42]. Since the structure of loud-calls is inherited and call adaptation forces a similar structure, gene flow could achieve the major influence on the structural variation of calls [15]. By combining the phylogenetic reconstruction of Meyer and colleagues [24] and the results of our study (Figures 2, 5A), we can observe a trend to simplification in call structure over time. However, it is difficult to explain why we found such a simplification in call structure. We cannot answer whether this is a general rule or whether this is a *Presbytis*-specific trait. Crested gibbons show an ambiguous result [15], where after a long period of syllable types with simple frequency modulation, a trend to a slightly more complex modulation appears. More acoustic comparisons with more species and at a higher taxonomic level are necessary to answer this question.

Interestingly, *P.potenziani *was regarded as most basal lineage [43] and due to similarities in call structure, the species was proposed as closely affiliated with *P.thomasi *[21]. However, neither is the case, since *P.potenziani *derived much later [24]. The specific call structure of *P.potenziani *is therefore either the result of an analogous evolution or a pleisiomorphic *Presbytis *feature. To clarify this issue further research is needed and particularly genetic and acoustic data on the Bornean and Malaysian taxa will help to better understand the evolution and phylogeography of the genus.

For instance, the call structure of *P.rubicunda *seems to be similar to *P.melalophos *calls [23] and, as discussed above, molecular genetic data also group *P.rubicunda *with *P.melalophos *[24]. This close relationship can partly help to explain the interesting feature of general allopatry of respective *Presbytis *taxa in Sumatra, and sympatry in Borneo [1]. *P.rubicunda *originated on Sumatra and subsequently invaded Borneo during the middle Pleistocene via a proposed connection between both islands [44]. At this time Borneo was already colonized by the Bornean species *P.chrysomelas, P.frontata and P.hosei*. As a result of this second colonization, *P.rubicunda *is sympatric today with the three other species wherever their ranges overlap [45].

## Conclusions

In this study we have shown that vocal similarity highly correlates with genetic relatedness; these two measures also correlate significantly with geographic distance, but the strength of the relationship is lower. Accordingly, acoustic analysis of surili loud-calls has been proven to be a promising and powerful tool to support taxon-affiliation and phylogenetic relatedness. In addition, we were able to confirm the proposed paraphyly of *P.melalophos *by differences in loud-call structure. Furthermore, acoustic analysis can be used as a tool to support proposed migration routes. These findings might also help to explain taxonomic relationships and migration backgrounds in other nonhuman primate taxa, as long as they have similar constraints in their vocal communication.

## Methods

### Survey locations and data collection

In 2007 and 2008 we conducted field surveys in 19 locations on Sumatra, Java and the Mentawai islands, and recorded male loud-calls of *P.thomasi, P.potenziani, P.melalophos *and *P.comata *(Figure 2). To find and track animals the field sites were explored between 5.30 am and 6 am until noon, and in the evening from 3 pm till sundown. When a group was encountered GPS data of the location (using a handheld GARMIn^© ^GPSMAP 76CSX), information about the group composition and the appearance of the animals (i.e. morphological characters, for instance pelage coloration or scars) were noted on data sheets whenever possible. All visual observations were made by using binoculars (8 × 32 Steiner Sky-Hawk).

Since surilis intensively respond to stranger call playbacks [35], we used a playback design to collect data under comparable conditions. Initially, vocalizations were opportunistically recorded to achieve a high quality call of each population. For the playback the quality of the recorded vocalizations was screened on a notebook using AVISOFT SASLAB Pro software version 5.1 (R. Specht, Berlin, Germany). Undisturbed calls from each population were selected and only one of these was used to stimulate response from respective study populations in the same area. At each site, we tried to avoid recording the same individuals by direct observations. Each playback comprised of 4 calls, which were played back one by one in 20 second intervals.

For the final data collection, playback treatments were amplified with a Vision David Speaker connected to a MP3-Player (Samsung YP-U3) from about 75 m distance of the focal group at a height of 2 m [35,46]. After the performance at least 15 minutes were recorded. If a response was given before the playback was finished the playback was stopped. To record vocalizations a solid state recorder (Mirant PMD 660 (Marantz, Japan); sampling rate: 44.1 kHz, 16 bit amplitude resolution) and a Sennheiser directional microphone (K6 power module, ME66 recording head, MZW66 pro windscreen, Sennheiser, Wedemark, Germany) were utilized. For each playback treatment the GPS position of the location, the group number, the date, time and the identity of a responding male were noted on data sheets.

### Acoustic analysis

Male surili loud-calls consist of iterations of single elements. *P.thomasi *and *P.potenziani *produce coughing elements at the beginning of the call. In *P.thomasi*, the successive elements rise in crescendo and increase in volume (see build up phase Figure 2), while the coughing elements in *P.potenziani *are equally loud and noisy. Both loud-calls end with howling tonal phrases including inhalation and exhalation elements (Figure 2). We considered these calls as completely developed when both parts were produced. *P.comata *loud-calls were considered as completely developed when a boost in loudness and frequency till the end of the call was present. *P.melalophos *loud-calls were considered as completely developed when they included at least 10 elements (the only two calls that were interrupted had less than 10 elements).

AVISOFT SASLAB Pro 5.1 was used to measure acoustic parameters and to generate spectrograms (FFT = 1024 pt, Frequency resolution = app. 27 Hz). To find the point with maximum energy at the beginning, ending and intermediate points of call elements in the spectrogram, the bounded reticule cursor tool of AVISOFT was used. To address different phases within loud-calls, each call was additionally divided into four quarters. Since all taxa produce exhalation elements, the amount of exhalation elements (Ex) was therefore divided by 4 and subsequently multiplied by 1, 2, 3 and 4, respectively. Odd numbers were rounded. If inhalation elements (In) were present; the second, third and fourth quarter always started with an exhalation element (for a detailed description of used parameters see Table 2 and Additional File 3).

**Table 2:** Description of acoustic parameters used in the analysis

Parameter Number	Parameter description
**1**	Duration of the entire call [s]: from the starting point of the first element till the ending point of the last element
**2**	Elements: amount of elements (inhalation and exhalation)
**3**	Elements per second [e/s]: amount of elements over the duration
**4**	Maximum frequency start [Hz]: maximum frequency of the starting points of the entire elements
**5**	Minimum frequency start [Hz]: maximum frequency of the entire starting points of elements
**6**	Maximum frequency end [Hz]: maximum frequency of the entire ending points of elements
**7**	Minimum frequency end [Hz]: minimum frequency of the entire ending points of elements
**8**	Mean frequency start [Hz]: arithmetic mean of the frequency of the entire starting points of elements
**9**	Mean frequency end [Hz]: arithmetic mean of the frequency of the entire ending points of elements
**10**	Exhalation elements: amount of exhalation elements
**11**	Inhalation elements: amount of inhalation elements
**12-15**	1^st ^-, 2^nd^-, 3^rd ^- and 4^th ^- quarter elements per second [e/s]: amount of elements over the duration of respective quarters
**16**	Middle part elements per second [e/s]: amount of elements over the duration of the 2^nd ^and 3^rd ^quarter
**17-20**	1^st ^-, 2^nd^-, 3^rd ^- and 4^th ^- quarter mean frequency start [Hz]: arithmetic mean of the frequency of the entire starting points of elements of respective quarters
**21**	Middle part mean frequency start [e/s]: arithmetic mean of the frequency of the entire starting points of elements of the 2^nd ^and 3^rd ^quarter
**22-23**	1^st ^-, and 2^nd ^- quarter mean frequency end [Hz]: arithmetic mean of the frequency of the entire ending points of elements of respective quarters

### Discriminant Function Analysis

For both Discriminant Function Analyses (DFAs), we excluded acoustic variables that could not be obtained in the majority of loud-calls. In the first DFA we used 23 acoustic parameters for 100 loud-calls from all 19 populations (Table 2, Figure 1). For the second DFA, including only the four *P.melalophos *subspecies (*melalophos, mitrata, sumatrana, bicolor*), we used the same 23 acoustic parameters for 71 loud-calls (population numbers 4-16, Figure 1). All acoustic parameters were conducted to stepwise DFAs in SPSS 19 [47]. The selection criterion for an acoustic parameter to be entered was p = 0.05 and p = 0.1 to be removed from the analysis. The assignment of loud-calls to the different populations was cross-validated by the leaving-one-out method [48], which involves leaving out each of the cases in turn, calculating the functions based on the remaining n-1 cases and then classifying the left-out case.

### Phylogenetic tree reconstruction

For reconstructing phylogenetic relationships of loud-call structure, we used the F values of pairwise distances of the stepwise DFA described above. These F values describe the pairwise similarity of the 19 populations in relation to their overall similarity. Based on these F values, a neighbor-joining tree of acoustic data was reconstructed in the program Neighbor of the PHYLIP package 3.69 [49]. The molecular-based phylogenetic tree derived from mitochondrial sequence data was redrawn from Figure 2 in [24] and shows only taxa included in the present study. Respective branch lengths refer to those obtained from the Bayesian reconstruction in [24].

### Correlation analysis between vocal structure, genetic and geographical distance

To test the statistical relationship between acoustic structure, and genetic and geographic distance matrices, we used a Mantel Test algorithm programmed in R (R. Mundry, Leipzig, Germany). For the analysis we only used populations where acoustic and genetic data was available (N = 17). The acoustic similarity matrices were generated as described above. Geographic coordinates were obtained via GPS and the geographic distance matrices were calculated from the minimum distance of different groups as implemented in GenAlEx 6.4.1 [50]. GenAlEx was also applied to calculate uncorrected pairwise genetic distances between haplotypes of a 1.8 kb fragment of the mitochondrial genome. Respective haplotypes were recently published by our group [24] (GenBank accession numbers: JF295100-JF295101 [*P.m.mitrata*], JF295104 [*P.m.melalophos*], JF295106-JF295109 [*P.m.bicolor*], JF295117-JF295118 [*P.comata*], JF295124-JF295125 [*P.thomasi*], JF295119-JF295121 [*P.potenziani*]).

## Authors' contributions

DM designed the study, collected acoustic and genetic samples, did laboratory work, analyzed data and wrote the paper. JKH designed the study and wrote the paper. DR collected acoustic data and supported fieldwork. AW collected acoustic and genetic samples and did laboratory work. CR designed the study, did laboratory work, analyzed data and wrote the paper. KH designed the study, analyzed data and wrote the paper. All authors read and approved the final manuscript.

## Supplementary Material

Additional file 1 **Calculated values of the arithmetic mean and the standard derivation of measured variables (pdf)**. Click here for file

Additional file 2 **Photographs of wild *Presbytis *taxa from Sumatra, Java and the Mentawai islands**. Numbers refer to locations in Figure 1 (photograph of *P.thomasi *by Cedric Buttoz Girard, all others by Dirk Meyer & Ambang Wijaya) (tif). Click here for file

Additional file 3 **Spectrogram of a *Presbytis *loud-call with examples for measured parameters (tif)**. Click here for file
