# Supplementary material for: Acoustic structure of male loud-calls support molecular phylogeny of Sumatran and Javanese leaf monkeys (genus Presbytis)
Source: BMC Evol Biol. 2012 Feb 6;12:16. doi: 10.1186/1471-2148-12-16 (PMC3295661; doi:10.1186/1471-2148-12-16)
Supplement: Additional file 1 — Calculated values of the arithmetic mean and the standard derivation of measured variables (pdf). [file 1471-2148-12-16-S1.PDF]

**Additional File 1**: Calculated values of the arithmetic mean and the standard derivation of measured variables.

| | | ***Presbytis comata*** | | | ***Presbytis m. mitrata*** | | | ***Presbytis m.melalophos*** | | | ***Presbytis m.bicolor*** | | | ***Presbytis m.sumatrana*** | | | ***Presbytis potenziani*** | | | ***Presbytis thomasi*** | | |
|---|---|---|---|---|---|---|---|---|---|---|---|---|---|---|---|---|---|---|---|---|---|---|
| **number** | **Parameter** | **arithmetic mean** | **standard derivation** | **n** | **arithmetic mean** | **standard derivation** | **n** | **arithmetic mean** | **standard derivation** | **n** | **arithmetic mean** | **standard derivation** | **n** | **arithmetic mean** | **standard derivation** | **n** | **arithmetic mean** | **standard derivation** | **n** | **arithmetic mean** | **standard derivation** | **n** |
| 1 | duration | 2.86 | .26 | 10 | 2.39 | .33 | 19 | 2.41 | .50 | 29 | 2.52 | .53 | 15 | 2.53 | .40 | 8 | 4.17 | .42 | 9 | 3.58 | .35 | 10 |
| 2 | Elements | 52 | 8 | 10 | 26 | 6 | 19 | 20 | 4 | 29 | 19 | 4 | 15 | 19 | 3 | 8 | 27 | 2 | 9 | 30 | 4 | 10 |
| 3 | elemente/s | 18.20 | 1.91 | 10 | 10.85 | 2.36 | 19 | 8.28 | 1.05 | 29 | 7.35 | .40 | 15 | 7.45 | .18 | 8 | 6.42 | .56 | 9 | 8.50 | 1.00 | 10 |
| 4 | f max s | 5537 | 1365 | 10 | 4732 | 623 | 19 | 5132 | 837 | 29 | 7587 | 2665 | 15 | 7006 | 945 | 8 | 1757 | 113 | 9 | 1752 | 226 | 10 |
| 5 | f min s | 759 | 322 | 10 | 2103 | 1089 | 19 | 2803 | 1164 | 29 | 4627 | 2328 | 15 | 4296 | 1368 | 8 | 517 | 454 | 9 | 409 | 65 | 10 |
| 6 | fmax e | 5604 | 1370 | 10 | 3665 | 1029 | 19 | 2429 | 821 | 29 | 2609 | 1132 | 15 | 1730 | 277 | 8 | 1779 | 155 | 9 | 1765 | 122 | 10 |
| 7 | fmin e | 1076 | 328 | 10 | 812 | 401 | 19 | 1160 | 324 | 29 | 1657 | 439 | 15 | 979 | 164 | 8 | 540 | 486 | 9 | 376 | 84 | 10 |
| 8 | fmean s | 1695 | 469 | 10 | 3429 | 869 | 19 | 4199 | 832 | 29 | 6484 | 2492 | 15 | 5837 | 788 | 8 | 1119 | 229 | 9 | 972 | 171 | 10 |
| 9 | fmean e | 2289 | 355 | 10 | 1715 | 303 | 19 | 1530 | 253 | 29 | 2096 | 792 | 15 | 1279 | 90 | 8 | 1115 | 271 | 9 | 993 | 86 | 10 |
| 10 | EX | 29 | 4 | 10 | 20 | 3 | 19 | 19 | 4 | 29 | 19 | 4 | 15 | 19 | 3 | 8 | 13 | 1 | 9 | 19 | 3 | 10 |
| 11 | IN | 24 | 4 | 10 | 6 | 5 | 19 | 1 | 2 | 29 | 0 | 0 | 15 | 0 | 0 | 8 | 13 | 1 | 9 | 12 | 2 | 10 |
| 12 | 1/4 t/e | .05 | .01 | 10 | .09 | .01 | 19 | .09 | .01 | 29 | .10 | .01 | 15 | .09 | .00 | 8 | .16 | .02 | 9 | .08 | .01 | 10 |
| 13 | 2/4 t/e | .04 | .01 | 10 | .09 | .02 | 19 | .10 | .01 | 29 | .12 | .01 | 15 | .11 | .00 | 8 | .21 | .02 | 9 | .11 | .02 | 10 |
| 14 | 3/4 t/e | .05 | .01 | 10 | .09 | .03 | 19 | .12 | .02 | 29 | .13 | .01 | 15 | .13 | .00 | 8 | .14 | .02 | 9 | .14 | .02 | 10 |
| 15 | 4/4 t/e | .07 | .02 | 10 | .11 | .04 | 19 | .14 | .02 | 29 | .14 | .01 | 15 | .16 | .01 | 8 | .10 | .01 | 9 | .12 | .02 | 10 |
| 16 | middle (2/4-3/4) t/e | .04 | .01 | 10 | .09 | .02 | 19 | .12 | .02 | 29 | .14 | .01 | 15 | .13 | .00 | 8 | .17 | .03 | 9 | .12 | .02 | 10 |
| 17 | 1/4 mean f start EX | 1316 | 196 | 10 | 3227 | 798 | 19 | 3566 | 794 | 29 | 5691 | 2425 | 15 | 5068 | 878 | 8 | 1445 | 106 | 9 | 1199 | 395 | 10 |
| 18 | 2/4 mean f start EX | 1394 | 221 | 10 | 3585 | 742 | 19 | 4132 | 833 | 29 | 6627 | 2698 | 15 | 5560 | 613 | 8 | 1554 | 93 | 9 | 1409 | 295 | 10 |
| 19 | 3/4 mean f start EX | 1504 | 186 | 10 | 3799 | 709 | 19 | 4434 | 893 | 29 | 6880 | 2695 | 15 | 6031 | 765 | 8 | 870 | 246 | 9 | 1076 | 319 | 10 |
| 20 | 4/4 mean f start EX | 3774 | 1103 | 10 | 4282 | 569 | 19 | 4849 | 798 | 29 | 6732 | 2386 | 15 | 6719 | 1038 | 8 | 449 | 14 | 9 | 495 | 54 | 10 |
| 21 | middle (2/4-3/4) mean f start EX | 1452 | 196 | 10 | 3692 | 719 | 19 | 4280 | 859 | 29 | 6741 | 2688 | 15 | 5803 | 678 | 8 | 1205 | 159 | 9 | 1250 | 304 | 10 |
| 22 | 1/4 mean f end EX | 1539 | 168 | 10 | 1571 | 294 | 19 | 1534 | 326 | 29 | 2206 | 1002 | 15 | 1400 | 234 | 8 | 1482 | 156 | 9 | 1274 | 411 | 10 |
| 23 | 2/4 mean f end EX | 1768 | 232 | 10 | 1244 | 404 | 19 | 1399 | 261 | 29 | 2143 | 1035 | 15 | 1292 | 161 | 8 | 1504 | 185 | 9 | 1508 | 198 | 10 |
