# Supplementary figures and images for: Acoustic structure of male loud-calls support molecular phylogeny of Sumatran and Javanese leaf monkeys (genus Presbytis)
Source: BMC Evol Biol. 2012 Feb 6;12:16. doi: 10.1186/1471-2148-12-16 (PMC3295661; doi:10.1186/1471-2148-12-16)

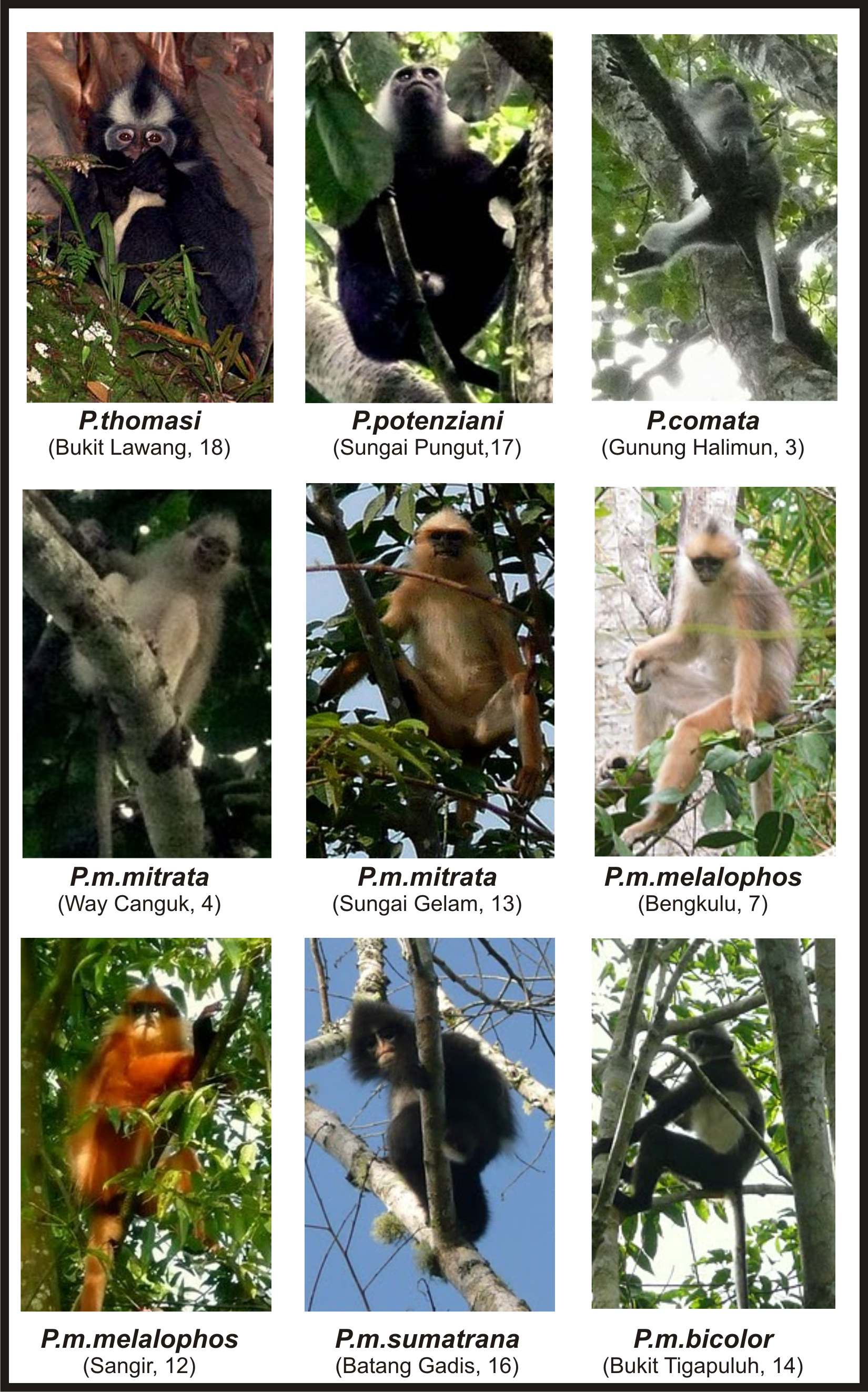

Supplement: Additional file 2 — Photographs of wild Presbytis taxa from Sumatra, Java and the Mentawai islands. Numbers refer to locations in Figure 1 (photograph of P.thomasi by Cedric Buttoz Girard, all others by Dirk Meyer & Ambang Wijaya) (tif). [file 1471-2148-12-16-S2.TIFF]

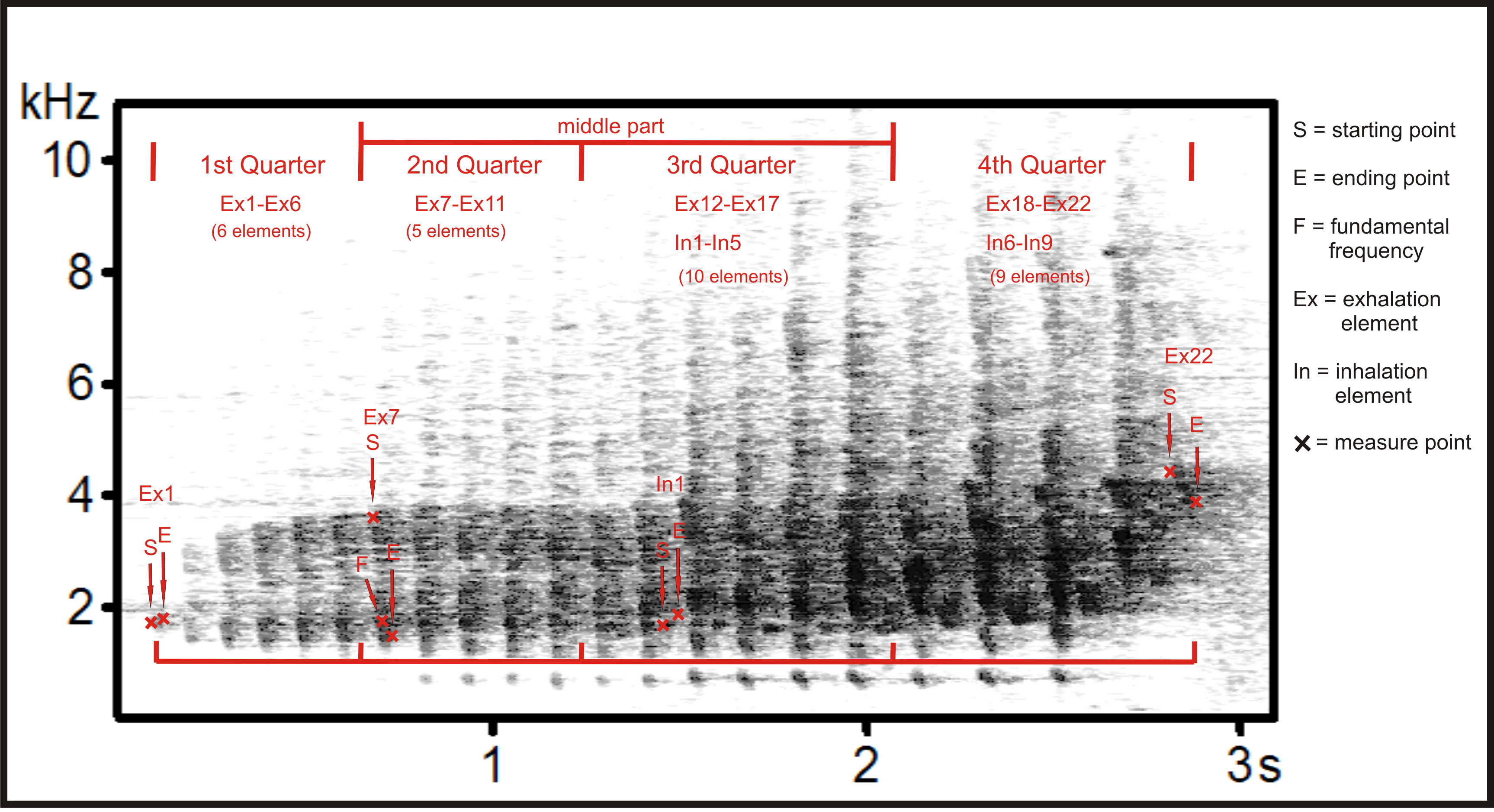

Supplement: Additional file 3 — Spectrogram of a Presbytis loud-call with examples for measured parameters (tif). [file 1471-2148-12-16-S3.TIFF]
